# MRI Overestimates Excitotoxic Amygdala Lesion Damage in Rhesus Monkeys

**DOI:** 10.3389/fnint.2017.00012

**Published:** 2017-06-08

**Authors:** Benjamin M. Basile, Chloe L. Karaskiewicz, Emily C. Fiuzat, Ludise Malkova, Elisabeth A. Murray

**Affiliations:** ^1^Section on the Neurobiology of Learning and Memory, Laboratory of Neuropsychology, National Institute of Mental Health, National Institutes of Health (NIH)Bethesda, MD, United States; ^2^Department of Pharmacology and Physiology, Georgetown University Medical CenterWashington, DC, United States

**Keywords:** amygdala, lesion, excitotoxic, ibotenic acid, T2 MRI, nonhuman primate

## Abstract

Selective, fiber-sparing excitotoxic lesions are a state-of-the-art tool for determining the causal contributions of different brain areas to behavior. For nonhuman primates especially, it is advantageous to keep subjects with high-quality lesions alive and contributing to science for many years. However, this requires the ability to estimate lesion extent accurately. Previous research has shown that* in vivo* T2-weighted magnetic resonance imaging (MRI) accurately estimates damage following selective ibotenic acid lesions of the hippocampus. Here, we show that the same does not apply to lesions of the amygdala. Across 19 hemispheres from 13 rhesus monkeys, MRI assessment consistently overestimated amygdala damage as assessed by microscopic examination of Nissl-stained histological material. Two outliers suggested a linear relation for lower damage levels, and values of unintended amygdala damage from a previous study fell directly on that regression line, demonstrating that T2 hypersignal accurately predicts damage levels below 50%. For unintended damage, MRI estimates correlated with histological assessment for entorhinal cortex, perirhinal cortex and hippocampus, though MRI significantly overestimated the extent of that damage in all structures. Nevertheless, ibotenic acid injections routinely produced extensive intentional amygdala damage with minimal unintended damage to surrounding structures, validating the general success of the technique. The field will benefit from more research into* in vivo* lesion assessment techniques, and additional evaluation of the accuracy of MRI assessment in different brain areas. For now, *in vivo* MRI assessment of ibotenic acid lesions of the amygdala can be used to confirm successful injections, but MRI estimates of lesion extent should be interpreted with caution.

## Introduction

Despite recent methodological advances in behavioral neuroscience with nonhuman primates, selective lesions are still the most definitive way to establish a causal relation between a brain area and a behavior. The state-of-the-art technique for making selective lesions is with injections of excitotoxins, such as ibotenic acid (see Murray and Baxter, 2006; Amaral, 2016 for reviews). Ibotenic acid causes selective degeneration of nerve cells around the injection site, without damage spreading to cell bodies of connected areas, causing degeneration of incoming nerve terminals, or affecting fibers of passage (Schwarcz et al., 1979). Because of the relatively selective nature of ibotenic acid lesions, they provide insights that are not possible with other lesion techniques (e.g., Rudebeck et al., 2013).

For nonhuman primates especially, it is advantageous to keep subjects with high-quality lesions alive and contributing to science for many years. Nonhuman primates, such as rhesus monkeys, are long-lived, allowing for unique longitudinal studies of brain plasticity following damage (Bachevalier et al., 2016; Bliss-Moreau et al., 2016). Monkeys are often tested on complex cognitive tasks that require long periods of training (e.g., Murray et al., 1993; Basile and Hampton, 2011; Basile et al., 2015). This acquired cognitive sophistication makes them particularly good models of human cognition, and thus for studying the effects of brain damage on cognition. Practically, monkeys are expensive research subjects, and having subjects participate in multiple studies maximizes scientific benefit, minimizes scientific cost, and helps achieve the three Rs of animal research. However, realizing these advantages requires the ability to accurately evaluate lesion quality *in vivo*.

The most common method of assessing lesion extent *in vivo* is with magnetic resonance imaging (MRI). In some areas, such as the hippocampus, the massive cell loss following injection of excitotoxins results in marked volume reduction, and damage can be quantified by comparing the volume of the hippocampus on T1-weighted MRI scans acquired before and after surgery. For this purpose, the postoperative scan should be acquired ~150 days after surgery, by which time the postoperative changes have stabilized (Málková et al., 2001). In the hippocampus, percent volume reduction accurately predicts the damage estimated from the traditional method of microscopic examination of Nissl-stained brain sections after histological processing of the tissue (Málková et al., 2001). Importantly, this correlation has been independently replicated (Nemanic et al., 2002). In both studies, the correlation between volume reduction and damage as assessed by histological examination was remarkably high (*r* = 0.95 in both cases).

More useful for researchers, hippocampal damage could also be accurately predicted just 6–11 days after surgery by quantifying the proportion of the hippocampus that was covered by white hypersignal, a sign of edema due to inflammation and cell death, in a T2-weighted MRI scan (Málková et al., 2001; Nemanic et al., 2002). Again, the correlations between damage extent estimated from hypersignal on MRI and from histological examination were high (*r* = 0.87 and 0.89, respectively). More importantly, the slope of the relation between MRI-based and histology-based estimates lay remarkably close to the true diagonal (see their Figure 7 in Málková et al., 2001). Similarly, Nemanic et al. (2002) also showed a relation close to the true diagonal and they noted that, in their population, T2 hypersignal provided a more accurate prediction than the T1 volume measure. Evaluation of lesions from T2 hypersignal has many advantages. First, as already mentioned, it accurately predicts hippocampal damage. Second, because the measure is available soon after the initial injections, it allows for follow-up surgeries to make additional injections if damage is incomplete (e.g., Hampton et al., 2004). Third, in many brain regions, hypersignal from T2-weighted scans is easier to assess than postoperative volume reduction. This is likely because the hippocampus is a special case, in that it is surrounded by ventricle. Consequently, evaluation of hypersignal on T2-weighted postoperative MR images has become the most widely used method of *in vivo* damage assessment for lesions based on injection of excitotoxins (e.g., Izquierdo and Murray, 2004; Chudasama et al., 2009; Maior et al., 2011; Rhodes et al., 2012; Raper et al., 2013; Rudebeck et al., 2013; Dal Monte et al., 2015; Costa et al., 2016; Weiss and Bachevalier, 2016).

Despite widespread use of T2 MRI to predict lesion extent, we have an incomplete idea of how well it predicts damage in brain areas other than the hippocampus. Málková et al. (2001) did find significant correlations for intended lesions of perirhinal cortex and parahippocampal cortex, but there were relatively few cases (six and four hemispheres, respectively) and the resulting damage was low (12% and 18%, respectively). They also found significant correlations between estimates of lesion volume based on T2 hypersignal and on histological examination for unintended damage to the amygdala, entorhinal cortex, area TE, and area TEO, but again the amount of damage was very low (9%, 11%, 2% and 2%, respectively), making it unclear how this translates to larger, intentional lesions. Thus, there is a clear need to evaluate how well T2-weighted MRI predicts large intentional lesions of brain areas other than the hippocampus.

Here, we evaluated the accuracy with which T2-weighted MRI predicts damage following ibotenic acid amygdala lesions in rhesus monkeys. We analyzed 19 hemispheres from 13 monkeys, using the same methods used previously (Málková et al., 2001). Because T2 hypersignal linearly predicts damage in the hippocampus (Málková et al., 2001; Nemanic et al., 2002), we hypothesized that it would also linearly predict damage in the amygdala.

## Materials and Methods

### Subjects

We analyzed 19 brain hemispheres from 13 male rhesus monkeys (mean age at first surgery: 6.5 years) who had participated in a variety of different neuropsychological studies. Prior to this analysis, monkeys were housed singly or in pairs, kept on a 12-h light-dark cycle, and had visual and auditory contact with conspecifics. Food and/or water were controlled as needed to maintain testing motivation, with their weight remaining above 85% of free-feeding weight. This study was carried out in accordance with the recommendations of the Guide for the Care and Use of Laboratory Animals and the US Animal Welfare Act. The protocol was approved by the NIMH Animal Care and Use Committee.

### Surgery

Surgical procedures for producing selective ibotenic acid lesions of the amygdala have been described previously (Málková et al., 1997; Murray and Mishkin, 1998; Meunier et al., 1999; e.g., Izquierdo and Murray, 2004; Izquierdo et al., 2005). Briefly, we planned injection sites by first obtaining T1-weighted structural MRI scans while monkeys were in a non-ferrous stereotaxic head frame. Injection locations were tailored to each individual amygdala, separated by ~2 mm in all planes, and comprised a mean of 19.7 sites (range = 15–28) per amygdala.

During surgery, monkeys were anesthetized with ketamine (100 mg/ml, 10 mg/kg, i.m.) and then maintained on isoflurane gas (1%–3% to effect). Blood pressure, respiratory rate, heart rate, temperature, blood oxygen saturation, and exhaled/inhaled CO_2_ were monitored throughout surgery. Using aseptic procedures, we opened the scalp in anatomical layers, removed a bilateral bone flap that covered all intended injections, and made small slits in the dura to allow needle penetration. For each needle track, we lowered a 30-gauge Hamilton syringe to the most ventral injection site, expressed 0.6–1.2 μl of ibotenic acid (10 mg/ml; Biosearch Technologies or Sigma) at 0.2 μl/min, waited 2 min to minimize backflow up the needle track, repeated for each subsequent injection site along the needle track, and then waited an additional 3 min before removing the needle from the brain. To reduce the risk of postoperative complications due to edema, bilateral lesions were carried out in two stages, separated by a minimum of 2 weeks. In all cases, the intended lesion included all nuclei of the amygdala. After the last injection, monkeys received mannitol (25%-20%, 30–37 ml, i.v., at 90–111 ml/h) to reduce brain swelling. Because these surgeries were completed over many years, the regimen for managing post-surgery pain, inflammation and infection differed slightly across monkeys as directed by veterinary staff, but typically included dexamethasone (4 mg/ml, i.m., 1.5 ml), cefazolin (330 mg/ml, 25 mg/kg, i.m.), ketoprofen (100 mg/ml, i.m., 0.1–0.2 ml), and ibuprofen (100 mg, p.o.).

### MRI Assessment

We matched individual images from the postoperative T2-weighted MRI scan at 1 mm intervals to individual drawings of a standard atlas of a rhesus monkey brain. Matching was done separately for each hemisphere, on the basis of local landmarks, and with reference to an atlas of the rhesus brain when necessary (Saleem and Logothetis, 2012). Image brightness and contrast were adjusted until gray and white matter were easily differentiated with neither being under- or over-exposed. We then plotted areas of hypersignal on the reference diagram, traced each plotted area in ImageJ (Schneider et al., 2012) using a digitizing tablet (Wacom, Kazo, Japan), and calculated the volume of the lesion by summing the area of the traced regions and multiplying by the slice thickness of 1 mm. This method normalizes the size of different brain regions for each monkey to that of the reference diagram and expresses damage as a proportion of the target structure. A subset of hemispheres (32%) were independently matched, plotted, traced, and measured by a second rater to assess inter-rater reliability. Estimated lesion volume was highly correlated between the two raters both for intended damage to the amygdala (Pearson’s correlation coefficient; *r*_4_ = 0.99, *p* < 0.001, *r*^2^ = 0.98) and for unintended damage to surrounding structures (entorhinal: *r*_4_ = 0.89, *p* = 0.017, *r*^2^ = 0.80; perirhinal: *r*_4_ = 0.95, *p* = 0.003, *r*^2^ = 0.90; hippocampus: *r*_4_ = 0.86, *p* = 0.030, *r*^2^ = 0.73).

### Histological Assessment

Following completion of their respective studies, animals were deeply anesthetized with sodium pentobarbital (100 mg/kg, i.p.) and perfused transcardially with aldehyde fixatives. The brain was then removed from the skull, cryo-protected, blocked in the coronal plane, and frozen in −80°C isopentane. Sections were cut in the coronal plane on a sliding microtome at a thickness of 50 μm and stored in ten parallel series. One or two series were mounted the following day on premade gelatin-coated slides, and then air dried. Sections were defatted, stained with thionine (1–3 min), rinsed with distilled water followed by ascending alcohol concentrations, and finally put into xylene and cover slipped with DPX mountant.

Following staining and mounting, we matched individual slides to individual drawings of coronal sections from the standard rhesus monkey brain at 1 mm intervals. Matching was done separately for each hemisphere, on the basis of local landmarks, and with reference to an atlas of the rhesus brain when necessary (Saleem and Logothetis, 2012). We then plotted areas of cell loss and gliosis on the standard sections and calculated the volume of the lesion in the same manner described above for the MR assessments. A subset of hemispheres (32%) were independently matched, plotted, traced, and measured by a second rater to assess inter-rater reliability. Quantification of total volume damaged was highly correlated between the two raters both for intended damage to the amygdala (Pearson’s correlation coefficient; *r*_4_ = 0.97, *p* = 0.001, *r*^2^ = 0.95) and for unintended damage to surrounding structures (entorhinal: *r*_4_ = 0.90, *p* = 0.014, *r*^2^ = 0.81; perirhinal: *r*_4_ = 0.94, *p* = 0.006, *r*^2^ = 0.88; hippocampus: *r*_4_ = 0.83, *p* = 0.039, *r*^2^ = 0.70).

### Data Analysis

We examined the correlation between assessment techniques using Pearson’s correlation coefficient, and the difference between the techniques using paired *t*-tests. We identified potential outliers using a cutoff of three standard deviations from the group mean followed by a leave-one-out analysis in which we tested whether effects remained significant with potential outliers removed. All tests were two tailed with *α* = 0.05. Effect sizes are *r*^2^ for correlations and Cohen’s d for *t*-tests (Hurlburt, 2006). Because our data were highly skewed towards ceiling and suffered from a restricted range, we arcsine transformed all proportions prior to statistical analysis to better approximate normality (Aron and Aron, 1999).

## Results

**Figure 1:**
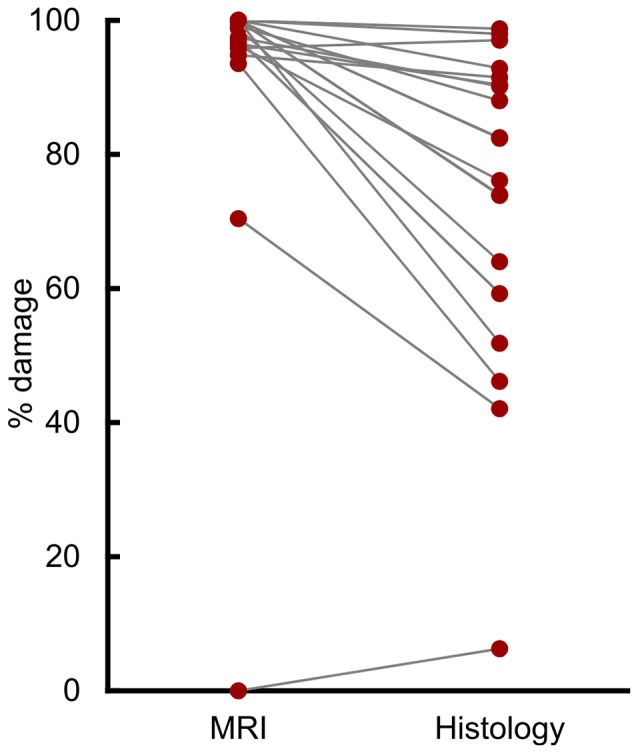
T2 magnetic resonance imaging (MRI) overestimated amygdala damage. Percent damage for each of 19 hemispheres as assessed by T2 MRI hypersignal and by histological examination.

Compared to histological examination, assessment from T2-weighted MRI scans significantly overestimated lesion volume (Figure 1; *t*_(18)_ = 5.31, *p* < 0.001, *d* = 1.21). The mean overestimation was 17.6%, with a maximum overestimation of 48.2%. Eight of the 19 hemispheres (42%) were overestimated by more than 20%, and 3 of 19 (16%) were overestimated by more than 30%.

**Figure 2:**
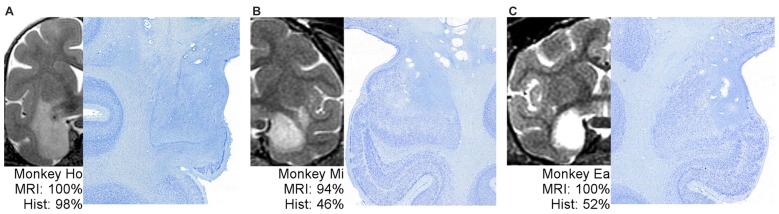
One example of well-predicted damage **(A)** and two examples of poorly-predicted damage **(B,C)**. For each monkey, a full hemisphere of the T2 MRI scan is shown on the left, with the white hypersignal indicating potential lesion extent. The matching histological section is shown on right at higher magnification to better view the border between large, dark, healthy cells and damaged tissue. The associated percentages indicate the coded damage level throughout the entire amygdala.

Figure 2 shows example T2 MR images next to photomicrographs of Nissl-stained material at matching levels. On the far left (Figure 2A) is an example of an MRI that predicted lesion volume very well. The MRI for Monkey Ho shows hypersignal completely covering the amygdala, extending through most of the entorhinal cortex but sparing the lower tip, just covering the top part of the perirhinal cortex, and extending into much of the adjacent portion of the claustrum. The lesion extent estimated using histological examination was remarkably consistent with the MRI assessment. In the middle and on the right (Figures 2B,C) are two examples of MRIs that predict lesion extent poorly. For Monkeys Mi and Ea, the MRI hypersignal appears to cover most or all of the amygdala, but histological examination showed that roughly half of the amygdala remained undamaged. Thus, the hypersignal in T2-weighted MRI scans is not necessarily a uniformly poor predictor of damage resulting from injection of excitotoxins; instead, it is an inconsistent predictor.

**Figure 3:**
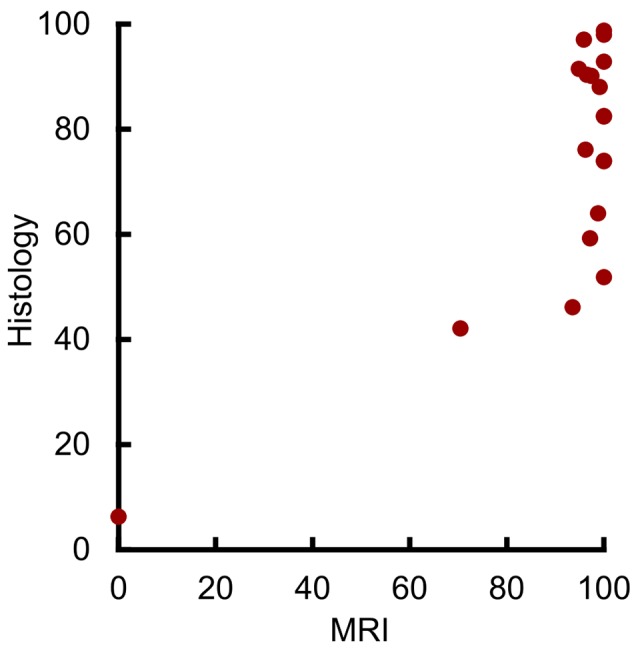
MRI hypersignal did not linearly predict amygdala damage. Percent of amygdala damage as estimated by T2 MRI and by histological examination. Each dot represents one hemisphere (*n* = 19).

Initial analysis showed a statistically significant correlation between the volume of amygdala lesions estimated from MRI and from histological examination (Figure 3; *r*_17_ = 0.73, *p* < 0.001, *r*^2^ = 0.53). However, our outlier analysis revealed that this effect was driven by a single outlying hemisphere (Figure 3, lowest left point). Compared to the remaining 18 hemispheres, the MRI-estimated damage was 14.1 standard deviations from the mean, and the leave-one-out analysis showed that omitting that data point abolished the linear correlation (*r*_16_ = 0.42, *p* = 0.082). Thus, the MRI-based estimates of amygdala lesion volume for the group as a whole could not be well described by a linear function.

Visual inspection suggested a possible threshold relation between damage as estimated by MRI and by histological examination, with a predictable linear relation at lower damage values. The majority of hemispheres showed near-complete MRI-estimated damage, despite estimates from histological examination showing extents between 50% and 100%. In contrast, the four hemispheres with the smallest damage estimate from both MRI and histological examination showed a significant linear correlation (*r*_2_ = 0.98, *p* = 0.019, *r*^2^ = 0.96). Taken together, this suggests that MRI may linearly predict damage levels below 50%, but that near-complete MRI-estimated damage can only grossly indicate that damage is at least 50%.

**Figure 4:**
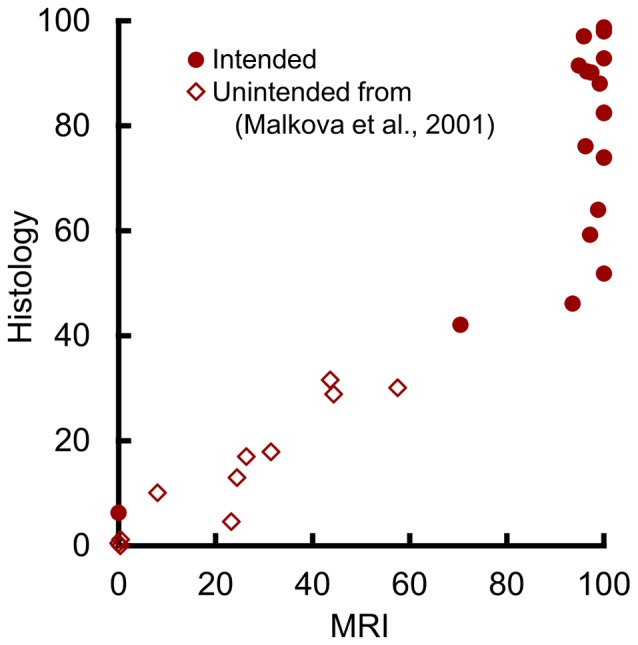
MRI hypersignal predicted amygdala damage in the 0%–50% range. Percent of amygdala damage as estimated by T2 MRI and by histological examination. Each dot represents one hemisphere. Solid circles (*n* = 19) represent intended damage in the current study, and open diamonds (*n* = 11) represent unintended damage from the hemispheres reported by Málková et al. (2001).

To assess the potential relation at lower damage levels, we plotted the values from 11 additional hemispheres with unintended amygdala damage following excitotoxic lesions of the hippocampus, parahippocampal cortex, or perirhinal cortex (Figure 4). These are the same estimates of unintentional amygdala damage reported in Málková et al. (2001), including all hemispheres that showed amygdala damage via either MR or histological assessment. The slopes of the regression lines from the four hemispheres with the lowest intentional damage, and from the 11 hemispheres with unintentional damage did not differ statistically (*t*_(11)_ = 1.49, *p* = 0.165). Nor was there a significant difference in group means when adjusted using slope as a covariate (*t*_(12)_ = 0.48, *p* = 0.639), suggesting that both intentional and unintentional amygdala damage were well described by the same regression line. Thus, amygdala damage in the range from 0% to 50% can be predicted from T2 hypersignal using the following equation.

% damage = (%MRI damage × 0.51) + 2.39

Unsurprisingly, amygdala lesion volume estimated by histological examination was linearly related to both the amount of ibotenic acid injected during surgery (*r*_16_ = 0.56, *p* = 0.017, *r*^2^ = 0.31) and to the number of injection sites (*r*_16_ = 0.50, *p* = 0.034, *r*^2^ = 0.25). These two measures are themselves highly correlated, but are not identical because each injection site used between 0.6 μl and 1.2 μl of ibotenic acid. However, these correlations both showed high variance, suggesting that some other factor is needed to explain individual differences in lesion extent.

**Figure 5:**
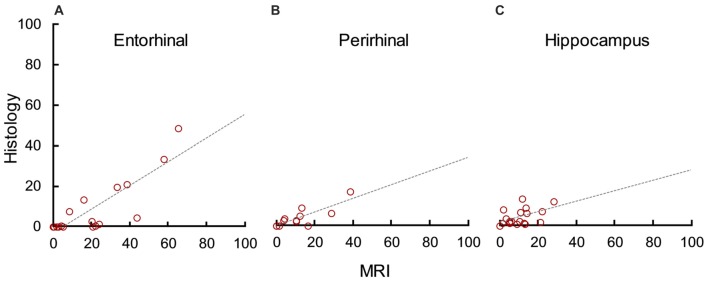
MRI hypersignal predicted but overestimated unintended damage to surrounding structures. Percent unintended damage to the entorhinal cortex **(A)**, perirhinal cortex **(B)** and hippocampus **(C)** as estimated by T2 MRI and by histological examination. Each dot represents one hemisphere (*n* = 19).

In addition to evaluating how well MRI predicted lesion completeness, we evaluated how well it predicted lesion selectivity by analyzing unintended damage to the entorhinal cortex (median = 1.40), perirhinal cortex (median = 0.00), and hippocampus (median = 2.12). MRI significantly overestimated damage to the entorhinal cortex (*t*_(18)_ = 5.55, *p* < 0.001, *d* = 1.27), perirhinal cortex (*t*_(18)_ = 3.11, *p* = 0.003, *d* = 0.71), and hippocampus (*t*_(18)_ = 4.23, *p* < 0.001, *d* = 1.35). However, there was a statistically significant correlation between damage estimates based on MRI and on histological examination in all three areas (Figure 5; entorhinal cortex: *r*_17_ = 0.83, *p* < 0.001, *r*^2^ = 0.69; perirhinal cortex: *r*_17_ = 0.86, *p* < 0.001, *r*^2^ = 0.73; hippocampus: *r*_17_ = 0.52, *p* = 0.023, *r*^2^ = 0.27). For all three areas, the slope of the linear regression line between MRI estimates and histological assessment more closely resembled that of the amygdala than previous findings in the hippocampus (compare Figures 4 and 5). This suggests that unintended damage to surrounding structures is also partially-predictable but overestimated by T2 hypersignal.

## Discussion

Contrary to our prediction, T2 MRI hypersignal overestimated amygdala damage following ibotenic acid lesions. It is not clear why amygdala lesions are less predictable than hippocampal lesions. Unlike the present findings with the amygdala, T2 MRI did not significantly over-predict hippocampal damage (Málková et al., 2001; Nemanic et al., 2002). Indeed, the regression line for hippocampal damage plotted by Malkova et al. lies remarkably close to the true diagonal (see their Figure 7 in Málková et al., 2001). One possible explanation is that the hippocampus is abnormally predictable because it is encapsulated by the ventricle. This encapsulation might contain the ibotenic acid during surgery, preventing it from diffusing into neighboring tissue at levels low enough to cause non-damaging inflammation that appears as hypersignal. In a similar vein, it is possible that the encapsulation contains the edema after surgery, preventing fluid from spreading to undamaged neighboring tissue and appearing as damage. A third possibility is that some unidentified aspect of the amygdala (e.g., receptor subtype distribution) makes it less reactive to excitotoxins. Regardless, our results do not dispute the predictive power of MRI for hippocampal lesions, but they do caution against generalizing those results outside of the hippocampus.

Our findings are consistent both with prior anecdotes about amygdala damage, and with the findings of unintentional damage from the two prior studies of hippocampal lesions. Anecdotally, Bauman et al. (2004b) sacrificed one monkey with an amygdala lesion early for health reasons and found that the damage seen during histological examination was less widespread than indicated by MRI. Similarly, Izquierdo and Murray (2004, 2010) reported extensive and uniform amygdala damage in 2004 when only T2 MRI was available to assess the damage, but they reported variable damage with partial sparing in 2010 when histology was available on the same group of monkeys. For systematic analyses, the unintended amygdala damage reported in Málková et al. (2001) confirms our current finding that MRI overestimates lesion volume, as their estimates lay along the regression line suggested by our hemispheres with the lowest intentional damage (Figure 4). Further, the unintended damage they saw in entorhinal cortex and area TE were also numerically, but not statistically, overestimated by MRI. Similarly, Nemanic et al. (2002) reported a nonsignificant trend for MRI to overestimate unintended damage to parahippocampal areas TH and TF. Others have expressed hesitation about using T2 hypersignal to estimate lesion extent in the amygdala (Bauman et al., 2004a,b). Our results partially validate this hesitation.

Our observed unintended damage to the entorhinal cortex, perirhinal cortex, and hippocampus contributes to the evidence that MRI hypersignal overestimates lesion extent. Though there is a linear relation between MRI and histology, the trajectories of the correlations suggest that only damage under 50% can be consistently predicted. Consistent with the MRI predictions of amygdala damage, T2 hypersignal significantly overestimated damage in all three untargeted areas. The overestimation of unintended damage in multiple areas suggests two conclusions. First, future evaluations of intentional damage in other regions will also show overestimation. Second, it is the hippocampus and not the amygdala that is abnormal in its damage predictability by MRI. In the hippocampus, the surrounding ventricle may act as a natural boundary, effectively holding ibotenic acid within the bounds of the hippocampus during intentional lesions and acting as a blockade during lesions to surrounding areas. This explanation, if true, would reconcile the previous findings of well-predicted intentional hippocampal damage (Málková et al., 2001; Nemanic et al., 2002) with the current finding of weakly-predicted unintentional hippocampal damage. The position of the hippocampus, encapsulated by ventricle, and the locus of the injection site could make intentional hippocampal damage abnormally predictable via MRI.

We were unable to eliminate the discrepancy between MRI and histology by manipulating the brightness and contrast of the T2 images. Prior to plotting the hypersignal, it is standard practice to adjust the brightness and contrast of the MR images to achieve good discriminability of gray matter and white matter, with neither being over- or under-exposed. After collecting these data, we selected several example hemispheres that showed particularly large discrepancies and systematically manipulated brightness and contrast in an attempt to identify ideal image settings to reduce this discrepancy. Although manipulating brightness and contrast did slightly change the borders of where an individual rater might draw the estimated lesion on the reference diagram, in no case did it produce MRI hypersignals that approached the level of damage seen in the Nissl-stained slides. See Supplementary Figure S1 for an example. Further, the high reliability between raters, who adjusted image settings individually, and the systematic relation at damage levels below 50% suggest that the MRI’s overestimation is not an epiphenomenon produced by image settings.

One caveat to our finding is that we cannot know if the cells in the spared amygdala tissue were fully functional. When examining histological material, the spared cells were visually indistinguishable from healthy cells. This was especially evident in the brains with unilateral lesions, for which the unoperated hemisphere was available for a direct comparison. In addition, zones of cell loss were circumscribed rather than graded, suggesting that the excitotoxin was fully effective within a given radius around the injection site, but not outside that radius. If these sharp borders do accurately mark the spread of the excitotoxin, then one would expect the spared zones to contain functional neurons. However, the functionality of a cell cannot be determined from Nissl-stained tissue alone; thus, it will likely be informative to conduct more in-depth immunohistochemistry on apparently spared tissue.

These findings highlight the need for better *in vivo* assessment techniques. Follow-up T1 MR scans performed more than 150 days after surgery accurately predict hippocampal damage (Málková et al., 2001; Nemanic et al., 2002). However, the amygdala does not reliably shrink in volume the way the hippocampus does, and others have suggested that T1 scans might over-estimate amygdala damage (Machado et al., 2008). Further, waiting more than 150 days to evaluate damage with T1 scans does not allow for the type of rapid assessment that can be used to plan additional surgeries if damage is incomplete. One possible assessment technique is to use PET imaging to measure disruptions in glucose metabolism after surgery. Another possibility is that visualizing the location and spread of injected fluid immediately after surgery by combining the ibotenic acid with an MRI contrast agent such as manganese might accurately predict eventual damage. It is also possible that other scan types, such as T2* or FLAIR, might produce more predictive visualizations of post-surgical edema. Regardless, the benefits of keeping nonhuman primates with selective lesions alive and contributing to research are large enough to justify future studies to identify more accurate *in vivo* lesion assessment techniques.

Despite the limited power of T2 MRI for predicting lesion extent, this study validates injections of ibotenic acid as a good method for producing selective amygdala damage. Across this relatively large sample of subjects, median amygdala damage was substantial (82.4%), and median unintended damage to the entorhinal cortex, perirhinal cortex, and hippocampus was minor (1.4%, 0.0%, and 2.1%, respectively). Ibotenic acid also spares fibers of passage (Schwarcz et al., 1979; Nemanic et al., 2002). This combination of effective and selective damage is the current state in a long history of active and constant improvement in scientific technique (Murray and Baxter, 2006; Amaral, 2016). Specifically, the transition from less-selective aspiration lesions to highly-selective excitotoxic lesions has repeatedly changed what we know about brain function (Murray, 1996; Rudebeck et al., 2013), and will likely continue to do so. Selective excitotoxic lesions are a powerful tool to test the causal contributions of different brain areas to behavior, and the current results represent one step in our continual process to evaluate, refine, and improve our methods.

In summary, findings from the present study point to four conclusions: (1) Ibotenic acid injections remain an effective technique of producing selective amygdala lesions; (2) T2 MRI can be used to broadly verify successful targeting and injection for amygdala lesions; (3) T2 MRI can be used to predict the extent of excitotoxic amygdala lesions if the predicted damage is below 50%; and (4) T2 MRI cannot reliably predict amygdala lesion volumes above 50%.

## Author Contributions

BMB, CLK, ECF and LM collected and analyzed the data. BMB, CLK and EAM wrote the article. All authors contributed to study design and interpretation.

## Conflict of Interest Statement

The authors declare that the research was conducted in the absence of any commercial or financial relationships that could be construed as a potential conflict of interest.
