# Supplementary material for: MRI Overestimates Excitotoxic Amygdala Lesion Damage in Rhesus Monkeys
Source: Front Integr Neurosci. 2017 Jun 8;11:12. doi: 10.3389/fnint.2017.00012 (PMC5462941; doi:10.3389/fnint.2017.00012)
Supplement: Supplementary file 1 [file Image_1.pdf]

Supplementary Material

MRI overestimates excitotoxic amygdala lesion damage in rhesus monkeys

Benjamin M. Basile*, Chloe L. Karaskiewicz, Emily C. Fiuzat, Ludise Malkova, and Elisabeth A. Murray

***Correspondence:** Benjamin M. Basile: benjamin.basile@nih.gov

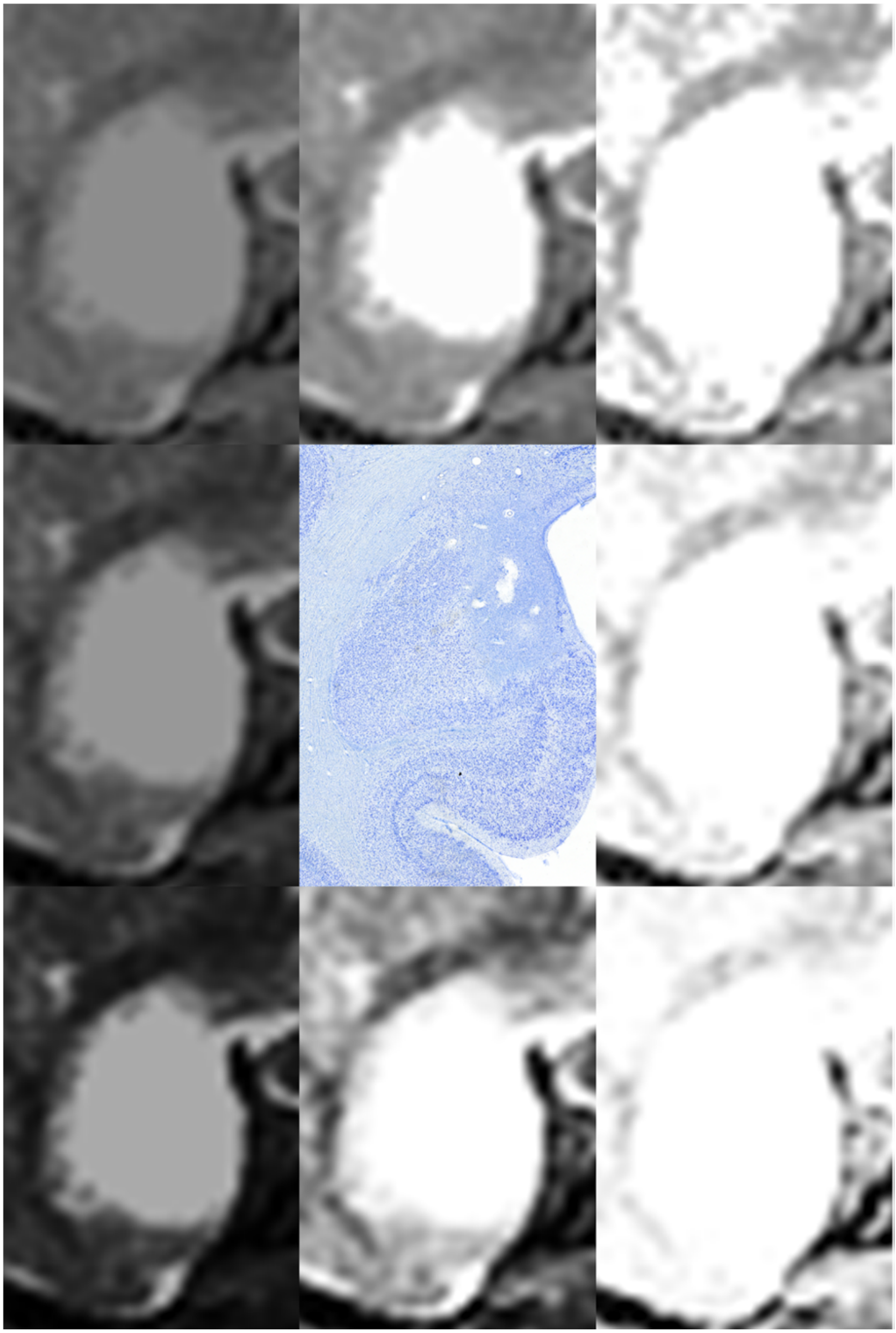

Supplementary Figure 1. Manipulating brightness and contrast does not eliminate the discrepancy between MRI and histology. Center: Nissl-stained slide showing the amygdala as predominantly spared. Surround: T2 MRI at different brightness (x-axis) and contrast (y-axis) levels, all showing the amygdala as predominantly damaged.
